# Infection‐induced seroconversion and seroprevalence of SARS‐CoV‐2 among a cohort of children and youth in Montreal, Canada

**DOI:** 10.1111/irv.13186

**Published:** 2023-08-25

**Authors:** Kate Zinszer, Katia Charland, Laura Pierce, Adrien Saucier, Marie‐Ève Hamelin, Margot Barbosa Da Torre, Julie Carbonneau, Cat Tuong Nguyen, Gaston De Serres, Jesse Papenburg, Guy Boivin, Caroline Quach

**Affiliations:** ^1^ School of Public Health University of Montreal Montreal Quebec Canada; ^2^ Centre for Public Health Research Montreal Quebec Canada; ^3^ Research Centre of Quebec‐Université Laval Quebec City Quebec Canada; ^4^ Ministère de la santé et des services sociaux Quebec City Quebec Canada; ^5^ National Institute of Public Health of Quebec Quebec City Quebec Canada; ^6^ Montreal Children's Hospital of the McGill University Health Centre Montreal Quebec Canada; ^7^ Research Centre of the Sainte‐Justine University Hospital Montreal Quebec Canada

**Keywords:** adolescents, Canada, children, SARS‐CoV‐2, seroconversion, seroprevalence

## Abstract

The EnCORE study is a prospective serology study of SARS‐CoV‐2 in a cohort of children from Montreal, Canada. Based on data from our fourth round of data collection (May–October 2022), we estimated SARS‐CoV‐2 seroprevalence and seroconversion. Using multivariable regression, we identified factors associated with seroconversion. Our results show that previously seronegative children were approximately 9–12 times more likely to seroconvert during the early Omicron‐dominant period compared to pre‐Omicron rounds. Unlike the pre‐Omicron rounds, the adjusted rate of seroconversion among 2‐ to 4‐year‐olds was higher than older age groups. As seen previously, higher seroconversion rates were associated with ethnic/racial minority status.

## 1 INTRODUCTION

SARS‐CoV‐2 transmission dynamics shifted dramatically in 2022 with the dominance of highly‐infectious Omicron variants and the loosening of preventative measures such as masking and social distancing.^1^ Disease surveillance systems had also changed, with PCR testing largely replaced by rapid antigenic testing of individuals along with population wastewater surveillance.^2^ In this context, longitudinal serological data could indicate the magnitude of infection‐induced SARS‐CoV‐2 seropositivity as well as determinants of seroconversion, which can help identify subpopulations that are vulnerable to infection. The EnCORE study is a prospective serology study of SARS‐CoV‐2 in children recruited from schools in four neighbourhoods in Montreal Canada. We present SARS‐CoV‐2 seroconversion and seroprevalence results from the fourth round of data collection (May to October 2022) and identify factors associated with the risk of seroconversion.

## 2 METHODS

As of October 2022, the EnCORE study comprised four rounds of data collection: Round 1 (October 2020 to April 2021, *N* = 1632), Round 2 (May to September 2021, *N* = 936), Round 3 (November 2021 to February 2022, *N* = 723) and Round 4 (May to October 2022, *N* = 726). Recruitment was re‐opened in Rounds 2 and 4. Participating children provided a dried blood spot (DBS) sample for serology testing, and parents provided household data through online questionnaires.^3^ Laboratory testing for seropositivity was based on enzyme‐linked immunosorbent assays using the receptor‐binding domain from the spike protein, the spike protein and the nucleocapsid protein, as antigens (Appendix S1).

In this analysis, we used Round 4 data, which included 726 participants aged 2 to 19 years old. Three hundred and ninety‐six of these children had been found seronegative in Round 3. We provide seroprevalence estimates for all 726 children in Round 4 and seroconversion rates between Rounds 3 and 4, for the 396 children that contributed to both rounds. Seroconversion was defined as the number of children seronegative in Round 3 and seropositive in Round 4. The seroconversion rate was the number of newly seropositive children in Round 4 divided by the sum of participants' time at risk between the third and fourth round DBS dates, in years. If a participant had a positive PCR or rapid antigen test, the test date (rather than the fourth round DBS date) marked the end of the participant's at‐risk time. To give context to the pandemic experience in Montreal and for our cohort of children through time, we present the Bank of Canada stringency index for Quebec, a percentage reflecting the strictness of public health measures, and for Rounds 2 to 4, the EnCORE cohort seroconversion rates and percentage of each age group that was vaccinated.

To assess the effect of each variable on the rate of seroconversion, we used multivariable quasi‐Poisson regressions after imputing data for missing values. To mitigate effects of loss to follow up, we applied inverse probability of censoring weights (Appendix S1). Unadjusted seroprevalence estimates for Round 4 by age, sex, vaccination status and prior seropositivity status were also estimated with correction by inverse probability of censoring weights. For further details on the study design and laboratory testing, please refer to Zinszer et al. and Zinszer et al.,^3^, ^4^, ^5^ and for the statistical methods, see Appendix S1.

## 3 RESULTS

**FIGURE 1:**
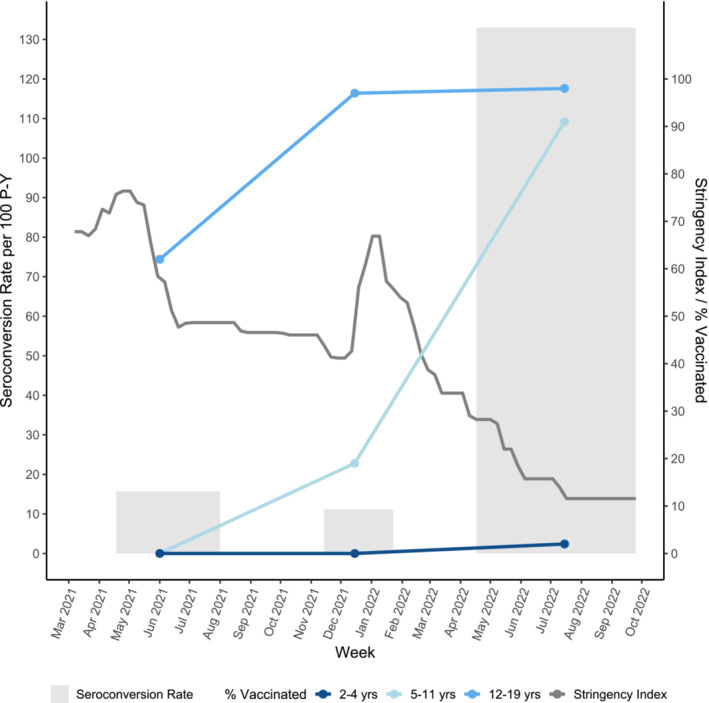
EnCORE cohort seroconversion rate per 100 person‐years (P‐Y) (light grey), percentage of Encore sample vaccinated with at least one dose, by age group (blues) and the Bank of Canada stringency index (%) for Quebec (dark grey) through Rounds 2, 3 and 4, with Omicron dominance starting in mid‐December 2021.

The infection‐induced seroconversion rate was 133 per 100 P‐Y (*N* = 396) among returning children, seronegative in Round 3; approximately 9–12 times that of pre‐Omicron rounds (Figure 1). Relative to the oldest age group, a 36% higher seroconversion rate was observed in 2‐ to 4‐year‐old children and a 24% lower rate in 5‐ to 11‐year‐old children (Table 1). This could largely be explained by age group differences in vaccination uptake. Vaccination was open to 12–19‐year‐olds in June 2021 and 98% were vaccinated prior to Round 4; 5–11‐year‐olds were eligible from November 2021 and 91% were vaccinated by Round 4, and the 2‐ to 4‐year‐olds could be vaccinated as of July 2022 and 2% were vaccinated by Round 4 (Figure 1). All vaccinated 2–4‐year‐olds had only one dose, over 83% of 5–11‐year olds had two or more doses, and over 86% of the 12–19‐year olds had two or more doses. Moreover, the percentage of the age groups having a vaccination within 120 days of their DBS was 2% for 2–4‐year‐olds, 32% for 5–11‐year‐olds and 27% for 12–19‐year‐olds, suggesting greater protection for the older participants. All 19 children that were previously seropositive in either Round 1 or 2 (but seronegative in Round 3), were found to be seropositive again in Round 4. Vaccinated children had a 57% decreased rate of infection‐induced seroconversion after adjusting for potential confounders; however, age group and vaccination were collinear in this model (Table 1). Females and children of parents identifying as an ethnic or racial minority also had a higher adjusted rate of seroconversion during this period than males and children of white parents, respectively. The unadjusted seroprevalence of our full sample of 726 children was 58.4% (95% CI 54.7%–62.0%) (Table 2).

**TABLE 1:** Crude seroconversion rates and adjusted seroconversion rate ratios, corrected for loss to follow‐up, for study population characteristics, estimated by multivariable quasi‐Poisson regression, for the fourth round of data collection (May to October 2022) of the EnCORE study.

	Seroconverted children (no. seropositive/no. total)	At‐risk person‐years	Crude seroconversion rate per 100 person‐years (95% CI)	Adjusted rate ratio (95% CI)	*p*‐Value
Overall	231/396	166.3	133.4 (116.2, 150.6)		
Sex					
Male	111/210	93.5	118.8 (96.7, 140.9)	1 [Reference]	
Female	120/186	79.8	150.3 (123.4, 177.2)	1.27 (1.03, 1.56)	0.027
Age, years					
2–4	33/43	17.9	184.4 (121.5, 247.3)	1.36 (1.04, 1.77)	0.025
5–11	88/171	76.4	115.2 (91.1, 139.2)	0.76 (0.61, 0.97)	0.024
12–18	110/182	79.0	139.2 (113.2, 165.3)	1 [Reference]	
BMI					
Underweight or normal weight	174/301	130.0	133.8 (114.0, 153.7)	1 [Reference]	
Overweight	41/69	30.8	133.1 (92.4, 173.9)	0.98 (0.76, 1.27)	0.88
Chronic medical conditions					
None	212/363	152.8	133.2 (115.3, 151.2)	1 [Reference]	
Yes	16/29	12.0	126.0 (64.2, 187.7)	0.94 (0.61, 1.45)	0.78
Parental respondent's level of education					
No bachelor's degree	45/75	32.2	139.8 (98.9, 180.6)	1 [Reference]	
Bachelor's degree	88/155	68.1	129.2 (102.2, 156.2)	0.92 (0.69, 1.22)	0.54
Graduate degree	94/160	70.0	134.3 (107.1, 161.4)	0.96 (0.73, 1.27)	0.77
Parental respondent's race and ethnicity					
White	194/345	152.4	127.3 (109.4, 145.2)	1 [Reference]	
Racial or ethnic minority	33/46	18.4	179.3 (118.2, 240.5)	1.44 (1.10, 1.89)	0.009
Bedroom density (people per bedroom)					
<1.5	156/269	102.0	132.3 (111.5, 153.0)	1 [Reference]	
1.5+	69/119	40.3	133.3 (101.8, 164.8)	1.00 (0.79, 1.26)	0.99
Annual household income (CAD)					
<100,000$	62/93	39.4	153.7 (115.5, 192.0)	1 [Reference]	
≥100,000$	136/237	96.8	133.4 (111.0, 155.8)	0.99 (0.76, 1.29)	0.94
Household member occupation					
Not essential	121/216	97.7	123.8 (101.7, 145.8)	1 [Reference]	
Essential, not health	62/98	42.9	144.6 (108.6, 180.6)	1.20 (0.94, 1.53)	0.14
Essential, health	45/79	31.4	143.3 (101.4, 185.2)	1.10 (0.82, 1.49)	0.51
Vaccination 10+ days before sample collection^†^					
No	52/61	24.6	211.7 (154.1, 269.2)	1 [Reference]	
Yes	179/335	148.7	120.4 (102.7, 138.0)	0.43 (0.32, 0.57)	<0.0001

^†^ Vaccinated with at least one dose of a COVID‐19 vaccine.

**TABLE 2:** Unadjusted seroprevalence, corrected for loss to follow‐up for study population characteristics for Round 4 (May to October 2022) of the EnCORE study.

	No. (%) seronegative	No. (%) seropositive	Seroprevalence (95% CI)	*p*‐Value
Total	303/726	423/726	58.4 (54.7, 62.1)	
Sex				
Female	132/359	227/359	63.1 (57.8, 68.1)	0.01
Male	171/367	196/367	53.9 (48.6, 59.0)	
Age, years				
2–4	26/105	79/105	76.8 (67.6, 83.9)	<0.0001
5–11	155/324	169/324	50.6 (45.1, 56.2)	
12–19	122/297	175/297	60.1 (54.3, 65.7)	
Vaccinated 10+ days before sample collection^†^				
No	27/146	119/146	81.7 (74.3,87.4)	<0.0001
Yes	276/580	304/580	52.7 (48.5, 56.9)	
Seropositive in a previous round				
No	197/440	243/440	55.9 (51.1, 60.6)	0.08
Yes	29/89	60/89	66.0 (55.0, 75.5)	

^†^ Vaccinated with at least one dose of a COVID‐19 vaccine.

## 4 DISCUSSION

Our study found that previously seronegative children were approximately 9–12 times more likely to seroconvert during data collection in the early Omicron‐dominant period (from May 2022 to October 2022) than in previous rounds (May to September 2021 and November 2021 to February 2022). A large‐scale cohort study in the United States found a 6–8‐fold increase in incidence rates between the Delta and Omicron waves in young children.^6^ The estimated seroprevalence of 58% was consistent with estimates from a repeated cross‐sectional seroprevalence study of Canadian blood donors, where infection‐induced seroprevalence was 46% in May 2022 and 67% in October 2022.^7^, ^8^ These results likely reflect Omicron's higher transmissibility combined with Québec's relaxation of public health measures.^9^, ^10^ As of May 2022 and the beginning of Round 4, masks were no longer mandatory in schools or other closed public spaces, with the exception of health‐care facilities and when using public transportation.^11^

Differences in seroconversion rates were observed by age group. Consistent with other studies, we found that young children were more affected by Omicron's surge with an estimated 36% higher rate of seroconverting, which was not seen in pre‐Omicron rounds.^4^, ^5^, ^6^ This may be explained by the high vaccine uptake in the older age groups. In the seroconversion cohort, only 2% of 2–4‐year‐olds were vaccinated and with only one dose. Older participants had a vaccine uptake exceeding 90%. Though older children may have been vaccinated over a year before their fourth round DBS, and BNT162b2 vaccine effectiveness against Omicron may wane significantly by 120 days,^12^ approximately one third of the older children had a second, third or fourth dose within 120 days of their DBS. This finding was consistent with CDC data demonstrating greater increases in seroprevalence between September and February 2022 among age groups with the lowest vaccination rates.^13^ We were not able to look at vaccination status in greater depth for our seroconversion analysis, given the multicollinearity with age groups, although this certainly warrants further exploration.

We found that having a parent self‐identify as a racial or ethnic minority was associated with a 44% higher rate of seroconverting during this period than children with white parents. This was also an important risk factor for seroconversion in EnCORE's earlier rounds and has been highlighted on multiple occasions since the beginning of the pandemic as a major risk factor of infection and infection severity.^4^, ^5^, ^14^ Our results support that even in the context of Omicron‐enhanced contagiousness, health inequalities still play a key role in SARS‐CoV‐2 incidence.

Two thirds of participants who were previously seropositive in any round were also seropositive in Round 4. All 19 children that were seropositive in the first or second round but seronegative in the third were again seropositive in Round 4. This could indicate a reinfection or the persistence of a detectable antibody response from an earlier infection. We previously found that the likelihood of remaining seropositive was 68% at 6 months versus 41% at 1 year; estimates vary in other studies of children and adults, with as many as 96% of children and 83% of adults having detectable antibodies at 11–12 months after infection.^5^, ^15^

A limitation of our study was the significant loss to follow‐up and based on anecdotal reports from parents, this was largely associated with the parent–child discomfort in providing the DBS. However, we corrected estimates using inverse probability of censoring weights and we found little evidence of differential patterns in loss to follow‐up.^5^ Nevertheless, the analysis was not powered to detect meaningful differences in seroconversion rates.

Despite these limitations, our results provide important insight into the changes in seroprevalence and seroconversion during the intense early Omicron phase of the pandemic. This information could be useful to assess transmission dynamics and evaluate the impact of the different interventions such as the removal of masking policies in schools or the availability of the COVID‐19 vaccines for young children. Although Omicron is a highly immune‐evasive variant, our data indicate that there is a protective effect related to vaccination, which warrants further exploration and supports the continued vaccination efforts, particularly in the most affected populations.

## AUTHOR CONTRIBUTIONS

*Conceptualization*: Kate Zinszer, Jesse Papenburg, Gaston De Serres and Caroline Quach. *Data curation*: Kate Zinszer, Katia Charland, Laura Pierce, Adrien Saucier, Margot Barbosa Da Torre, Jesse Papenburg, Marie‐Ève Hamelin, Julie Carbonneau, Cat Tuong Nguyen, Guy Boivin and Gaston De Serres. *Formal analysis*: Katia Charland. *Funding acquisition*: Kate Zinszer, Gaston De Serres and Caroline Quach. *Project administration*: Kate Zinszer and Laura Pierce. *Supervision*: Kate Zinszer, Katia Charland, Laura Pierce, Guy Boivin and Gaston De Serres. *Writing—original draft*: Kate Zinszer, Katia Charland, Laura Pierce and Adrien Saucier. *Writing—review and editing*: Kate Zinszer, Katia Charland, Laura Pierce, Adrien Saucier, Margot Barbosa Da Torre, Jesse Papenburg, Marie‐Ève Hamelin, Julie Carbonneau, Cat Tuong Nguyen, Guy Boivin, Gaston De Serres and Caroline Quach. Kate Zinszer had full access to all of the data in the study and takes responsibility for the integrity of the data and the accuracy of the data analysis.

## CONFLICT OF INTEREST STATEMENT

JP reports grants from MedImmune, grants and personal fees from Merck and AbbVie, and personal fees from AstraZeneca, all outside the submitted work.

## DATE AVAILABILITY STATEMENT

Data are available on reasonable request from the corresponding author.

## ETHICAL APPROVAL STATEMENT

The research ethics boards of the Université de Montréal and the Centre Hospitalier Universitaire Sainte‐Justine approved this study in accordance with the laws of the Government of Quebec. All parents and children gave consent prior to participation.

## Supporting information


**Appendix S1.** Supplementary Methods.
