# Supplementary material for: Infection‐induced seroconversion and seroprevalence of SARS‐CoV‐2 among a cohort of children and youth in Montreal, Canada
Source: Influenza Other Respir Viruses. 2023 Aug 25;17(8):e13186. doi: 10.1111/irv.13186 (PMC10457549; doi:10.1111/irv.13186)
Supplement: Supplementary file 1 — Appendix S1. Supplementary Methods. [file IRV-17-e13186-s001.docx]

**Appendix: Supplementary Methods**

**Serostatus determination**

If a participant was positive for any two of the three antigens, receptor-binding domain of the spike protein (RBD), the spike protein (S) or the nucleocapsid protein (N), and was unvaccinated or received their first dose of a COVID-19 vaccine within 9 days of their dried blood spot (DBS) sample, they were classified as being infection-induced seropositive ^1^ . If a participant received at least one dose of a COVID-19 vaccine at least 10 days prior to collecting their DBS sample and was positive for N and at least one other antigen, they were classified as being infection-induced seropositive. Those who were RBD and S positive but N negative, at least 10 days from the date of their first vaccination, were classified as vaccine-induced seropositive but seronegative for SARS-CoV-2 infection. Regardless of vaccination status, participants negative for all antigens were classified as seronegative for SARS-CoV-2 infection and participants negative for two antigens were classified as inconclusive.

**Statistical methodology**

Seroprevalence was defined as the proportion of children that were seropositive following a SARS-CoV-2 infection among children providing a DBS sample of adequate quality.

The seroconversion analyses included only children that had participated in serology testing in the third and fourth rounds of data collection and were seronegative in the third round. We present the unadjusted seroconversion rates for Round 4 by study participant characteristics. The seroconversion rate was the number of newly seropositive participants divided by time at risk between rounds 3 and 4. At-risk person-time for each participant was calculated as the time between the date of DBS in the third round and the fourth round. If a positive PCR or antigen test had occurred between the 3^rd^ and 4^th^ Round in a seropositive child at Round 4, the participant’s time at risk ended at that PCR/antigen test date.

To estimate the adjusted seroconversion rate ratios, we used multivariable quasi-Poisson regression. The log-transformed follow-up time at risk of infection, i.e., time from last negative serology test, was an offset in the model. For each independent variable, the minimally sufficient adjustment set was identified from a directed acyclic graph and was used in the regression model for that independent variable. As a large proportion of children were lost to follow up, inverse probability of censoring weights (IPCW), estimated by the ipw package in R, were applied to mitigate any resulting selection bias. Robust standard errors were computed using the sandwich package in R. Multivariate Imputation by Chained was carried out since ~17% of household income and 7% of BMI percentile values were missing (R package mice). All other variables had no more than 2% missingness. Results include the seroconversion rate ratios, 95% confidence intervals and p-values based on the IPCW weighted and imputed data.

Seroprevalence estimates weighted by IPCW are presented by child and household characteristics along with p-values from chi-square tests (ipw and survey package in R). All analyses were carried out using R version 4.2.0.

**References**

1. Kanji JN, Bailey A, Fenton J, et al. Detection of SARS-CoV-2 antibodies formed in response to the BNT162b2 and mRNA-1237 mRNA vaccine by commercial antibody tests. Vaccine. 2021;39(39):5563-5570. doi:10.1016/j.vaccine.2021.08.022
